# Expression Pattern of Leptin and Its Receptors in Endometrioid Endometrial Cancer

**DOI:** 10.3390/jcm10132787

**Published:** 2021-06-24

**Authors:** Dariusz Boroń, Robert Nowakowski, Beniamin Oskar Grabarek, Nikola Zmarzły, Marcin Opławski

**Affiliations:** 1 Department of Histology, Cytophysiology and Embryology, Faculty of Medicine, University of Technology in Katowice, 41-800 Zabrze, Poland; nowakowskirobert926@gmail.com (R.N.); bgrabarek7@gmail.com (B.O.G.); nikola.zmarzly@gmail.com (N.Z.); 2 Department of Gynecology and Obstetrics with Gynecologic Oncology, Ludwik Rydygier Memorial Specialized Hospital, 31-826 Kraków, Poland; marcin.oplawski@gmail.com; 3 Departament of Gynecology and Obstetrics, TOMMED Specjalisci od Zdrowia, Fredry 22, 40-662 Katowice, Poland; 4 BOG-JET BENIAMIN OSKAR GRABAREK Company, Trzebinska 3/31, 32-500 Chrzanów, Poland; 5 Department of Nursing and Maternity, High School of Strategic Planning in Dabrowa Gornicza, 41-300 Dabrowa Goórnicza, Poland

**Keywords:** leptin, leptin receptor, endometrial cancer, obesity, expression profile

## Abstract

The identification of novel molecular markers and the development of cancer treatment strategies are very important as cancer incidence is still very high. Obesity can contribute to cancer progression, including endometrial cancer. Adipocytes secrete leptin, which, when at a high level, is associated with an increased risk of cancer. The aim of this study was to determine the expression profile of leptin-related genes in the endometrial tissue samples and whole blood of patients. The study material included tissue samples and whole blood collected from 30 patients with endometrial cancer and 30 without cancer. Microarrays were used to assess the expression profile of leptin-related genes. Then, the expression of leptin (LEP), leptin receptor (LEPR), leptin receptor overlapping transcript (LEPROT), and leptin receptor overlapping transcript-like 1 (LEPROTL1) was determined by the Real-Time Quantitative Reverse Transcription Reaction (RT-qPCR). The serum leptin concentration was evaluated using Enzyme-linked immunosorbent assay (ELISA). Leptin and its receptors were overexpressed both at the mRNA and protein levels. Furthermore, there were strong positive correlations between leptin levels and patient Body Mass Index (BMI). Elevated levels of leptin and its receptors may potentially contribute to the progression of endometrial cancer. These observations may be useful in designing endometrial cancer treatment strategies.

## 1. Introduction

Leptin is an adipokine, secreted by adipocytes, whose main task is to regulate energy balance, which is possible by lowering the appetite. It is also expressed in skeletal muscles, placenta [1], ovaries, stomach, and bone marrow [2]. Leptin acts by binding to the leptin receptor (LEPR), leptin receptor overlapping transcript (LEPROT), and leptin receptor overlapping transcript-like 1 (LEPROTL1) [3]. Interestingly, obese patients have a high concentration of serum leptin, which indicates that there is a resistance mechanism to this adipokine so that its high level does not prevent disturbances in the energy balance [4]. These elevated levels of leptin are associated with an increased risk of cancer, including kidney cancer, endometrial cancer [5], and breast cancer [6]. Leptin was found to stimulate processes favorable to tumor growth, its migration, and invasion. These include proliferation, inflammation, angiogenesis, as well as inhibition of tumor cell apoptosis [7]. In addition, leptin is secreted by tumor cells that also overexpress its receptors [8].

Endometrial cancer (EC) is one of the most frequently diagnosed gynecological cancers in the world. In 2020, EC was the sixth most common cancer in women. There were over 600,000 new cases and just under 100,000 deaths from endometrial cancer in 2020 [9]. EC mostly affects postmenopausal women, however, up to 25% of cases are diagnosed before menopause [10]. The main risk factors include obesity, diabetes, unopposed estrogen therapy, tamoxifen, polycystic ovary syndrome (PCOS), and nulliparity [11].

There are several endometrial cancer classification systems that allow for the assessment of the disease advancement. Two types of EC can be distinguished according to Bokhman. Type I accounts for approximately 80% of all endometrial cancer cases and has a good prognosis. It consists of low grade endometrioid ECs that are estrogen-dependent. Type II mainly includes non-endometrioid ECs with poor prognosis and high grade [12]. In addition to the well-known division based on staging [13] and grading [14], four subgroups are distinguished according to the genetic features: polymerase epsilon (POLE)-ultramutated, microsatellite instability-hypermutated, copy number low, and copy number high [15]. The goal of the Cancer Genome Atlas (TCGA) molecular groups was to eliminate over- or under-treatment by increasing the precision of diagnostics. Histological factors affect each group differently, which in turn is important for the assessment of their prognostic value. It is believed that the combination of molecular and histological characteristics could help in developing a new prognostic risk stratification in endometrial cancer patients [16].

Emerging classification systems and their updates show the need to further investigate the processes underlying endometrial cancer. It is important to keep in mind that molecular changes occur earlier than phenotypic ones, therefore the identification of new biomarkers may allow for early diagnosis of the disease and taking appropriate measures. Additionally, a more detailed understanding of cancer processes can help in developing an effective treatment strategy [17].

The aim of this study was to determine the expression profile of leptin-related genes in endometrial cancer tissue samples and whole blood of patients.

## 2. Materials and Methods

This study was approved by the Bioethical Committee operating at the Regional Medical Chamber in Krakow (185/KBL/OIL/202 and 186/KBL/OIL/2020). All procedures involving human participants were performed in accordance with the guidelines of the 2013 Declaration of Helsinki. Patient identifying information was removed prior to database analysis. The confidentiality of the data and the anonymity of the patients were maintained at all times. Informed consent was obtained from all subjects involved in the study. A written informed consent was collected and is kept within the patients’ medical records.

### 2.1. Patients

The study involved 60 women qualified for hysterectomy: 30 with diagnosed endometrioid endometrial cancer (study group) and 30 without neoplastic changes (control group), treated at the Department of Gynecology and Obstetrics with Gynecologic Oncology at the Ludwik Rydygier Memorial Specialized Hospital. Endometrial cancer was diagnosed based on a histopathological examination. All EC patients underwent radical removal of the uterus and the pelvic and pre-aortic lymph nodes.

The exclusion criteria from the study group included: non-endometrioid endometrial cancer, endometriosis or adenomyosis, adenocarcinoma with squamous elements, coexisting cervical cancer, history of other cancer types, use of hormone therapy 24 months prior the surgery, and extreme obesity (BMI > 40). The study and control groups included patients over 45 years of age and after childbearing period. Histopathological evaluation divided EC samples according to the degree of histological differentiation: G1, 15 cases; G2, 8 cases; and G3, 7 cases.

Endometrial tissue samples were collected during hysterectomy and placed in Eppendorf tubes containing Allprotect Tissue Reagent (Qiagen, Cat No./ID: 76405). The samples were then stored according to the manufacturer’s instructions. In addition, whole blood was collected from patients using PAXgene Blood RNA Tubes, which were stored at −20 °C until molecular analysis.

### 2.2. RNA Isolation

Total RNA was extracted from endometrial tissues with TRIzol reagent (Invitrogen Life Technologies, Carlsbad, CA, USA, Cat No. 15596026) according to the manufacturer’s instructions. RNA extraction from whole blood was performed using the PAXgene Blood RNA Kit (Qiagen, Cat No./ID: 762174) as recommended by the manufacturer.

The quality of the obtained extracts was assessed with agarose electrophoresis, and the concentration and purification by spectrophotometry. The value of the absorbance ratio 260/280 nm in the range 1.8–2.0 allowed for the qualification of the extract for microarray analysis.

### 2.3. mRNA Microarrays

The expression profile of leptin-related genes was determined with HG-U133A 2_0 oligonucleotide microarrays (Affymetrix, Santa Clara, CA, USA), the GeneChip™ 3′IVT PLUS Reagent Kit, and the GeneChip™ HT 3′IVT PLUS Reagent Kit (ThermoFisher, Cat No. 902416). The Affymetrix NetAffx™ Analysis Center database (http://www.affymetrix.com/analysis/index.affx; accessed on 1 October 2020) was used to acquire the probe names and their identification numbers after entering the phrase “leptin”. A GeneArray scanner (Agilent Technologies, Santa Clara, CA, USA) for microarray scanning was used to analyze the obtained data.

### 2.4. Real-Time Quantitative Reverse Transcription Reaction

In order to validate the results of the microarray experiment, Real-Time Quantitative Reverse Transcription Reaction (RT-qPCR) was performed. The expression profile of LEP, LEPROT, LEPROTL1, LEPR was determined using the SensiFast ™ SYBR No-ROX One-Step Kit, (Bioline, London, UK). β-actin (ACTB) was the endogenous control (Table 1).

The thermal profile of the reaction included: reverse transcription (45 °C, 10 min), polymerase activation (95 °C, 2 min), and 40 cycles including denaturation (95 °C, 5 s), annealing (60 °C, 10 s), elongation (72 °C, 5 s).

### 2.5. ELISA

Leptin protein levels were determined by an ELISA assay with the Human Leptin solid-phase ELISA KIT (Life Technologies Corporation, Invitrogen, Carlsbad, CA, USA; Cat No. KAC2281). The procedures were carried out in accordance with the manufacturer’s recommendations. The generated standard curve allowed for the assessment of leptin concentration in the analyzed samples.

### 2.6. Statistical Analysis

The Transcriptome Analysis Console programs (Thermo Fisher Scientific, Waltham, MA, USA) and Statistica 13.0 PL software (Cracow, Poland) were used to conduct statistical analysis. ANOVA and Tukey’s post hoc test were performed (*p* < 0.05). The results of changes in gene expression were shown as fold change (FC) compared to the control. The Pearson correlation test was conducted to investigate the relationship between patient characteristics and serum leptin levels.

## 3. Results

### 3.1. Characteristics of Patients Included in the Study and Control Group with the Analysis of the Differences between Groups

Table 2 shows the patient characteristics, including age, height, weight, and BMI, described as mean ± standard deviation (*p* < 0.05).

The statistical analysis showed the statistically significant differences in weight and BMI between analyzed groups (*p* < 0.05).

### 3.2. Leptin-Related Gene Expression Profile in Endometrial Tissues and Whole Blood of Patients Determined by Microarrays and RT-qPCR

A one-way ANOVA with the Benjamini-Hochberg correction showed that out of 38 mRNAs associated with leptin, the expression of 16 mRNAs significantly changed in endometrial cancer tissue samples compared to the control. Tukey’s post hoc test revealed that the number of mRNAs differentiating each grade from the control was as follows: G1 vs. C, 6 mRNAs; G2 vs. C, 6 mRNAs; and G3 vs. C, 4 mRNAs (*p* < 0.05).

Similarly, in whole blood samples, 15 out of 38 mRNAs significantly changed their expression in samples collected from EC patients compared to the control group. Tukey’s post hoc test indicated that the number of differentiating mRNAs was: G1 vs. C, 6 mRNAs; G2 vs. C, 6 mRNAs; and G3 vs. C, 3 mRNAs (*p* < 0.05).

**Figure 1:**
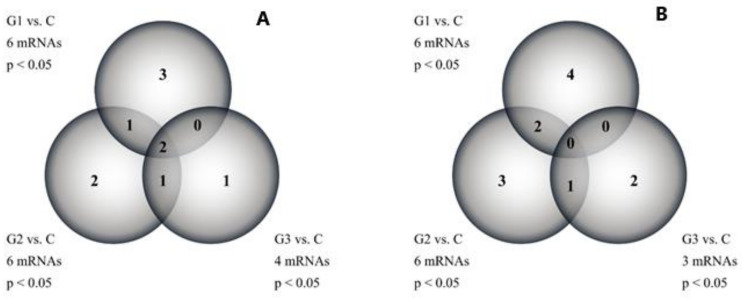
Venn diagram of the results obtained with the microarray method. (**A**)—tissue samples; (**B**)—whole blood; C, control; and G, endometrial cancer grade; *p* < 0.05 vs. C group; mRNA—messenger RNA.

In both cases, a Venn diagram was constructed to show genes characteristic of a given grade and common to several groups (Figure 1).

The results demonstrated that LEPR expression was significantly altered in all grades of endometrial cancer compared to control in both tissue and whole blood samples. Similarly, the LEP gene was characteristic of G2 and G3 cancers in both cases. On the other hand, LEPROTL1 was characteristic of G2 cancer tissue samples and G1 cancer in whole blood. Interestingly, LEPROT was observed to differentiate endometrial cancer samples regardless of its grade, while in blood samples it was a gene characteristic of G1 cancer.

In the next step, the expression profile of LEP, LEPROT, LEPROTL1, and LEPR in endometrial tissues and in the whole blood of patients was assessed using the RT-qPCR. Table 3 summarizes the results of the microarray and RT-qPCR experiments in the studied material, assuming the cut-off point of the fold-change (FC) values was > 3 or < −3. The exact *p*-values are shown in the supplementary material (Table S1).

The performed analysis revealed that the studied genes were overexpressed in endometrial cancer samples and whole blood collected from EC patients compared to the control group. This result was obtained with both microarrays and RT-qPCR.

### 3.3. The Level of Leptin Protein in the Serum of Patients Determined by ELISA

Protein expression was assessed in the serum of endometrial cancer patients and the control group using ELISA. The Pearson correlation test was also performed, which showed a significant relationship between BMI and leptin concentration (Table 4).

The conducted analysis showed that leptin expression increased with the grade of endometrial cancer. The observed overexpression at the protein level was consistent with the results obtained at the gene level. Additionally, strong positive correlations were found between BMI and the study groups, with the strength increasing with the grade of endometrial cancer.

## 4. Discussion

The development of molecular techniques has significantly contributed in recent years to the discovery of new treatment strategies and the identification of novel molecular markers [18]. This is particularly important in the case of cancer as the number of reported cases is still high, therefore further research is needed to develop new management strategies [19].

Obesity is currently a major health problem as it not only promotes the development of metabolic diseases and cardiovascular disease, but also contributes to cancer progression [20]. It is estimated that obesity in women worldwide in 2025 will be around 21%. These estimates are worrying as half of all female cancers are related to obesity [21]. These include endometrial cancer, the incidence of which has increased over the past 20 years, as has the percentage of obese women. Obesity is often associated with the coexistence of other health problems that may increase the risk of complications during hysterectomy and possible radiotherapy or chemotherapy [22]. About 10% of women cannot undergo standard surgery. Radiotherapy seems to be a promising option, however in obese women the recurrence rates are 18%. In addition, there are often dosimetric and technical difficulties, which means that radiotherapy cannot be used. Therefore, there is a great need to search for therapeutic methods for women at high surgical risk [23].

The relationship between obesity and cancer development is an important topic, as better understanding of the mechanisms contributing to cancer progression can help in the discovery of new methods of early diagnosis and effective treatment, with minimal risk [24]. Adipose tissue is very endocrine active and the increase in visceral fat affects its functions [25]. Moreover, large amounts of cytokines, such as tumor necrosis factor-α (TNF-α), interleukin-6 (IL-6), and leptin are produced [5], which can promote the recruitment of macrophages and impair the function of adipocytes, which ultimately induces chronic inflammation [26].

The activity of cancer cells is influenced by the tumor microenvironment, in which there are adipocytes and fibroblasts, which can secrete various cytokines [27]. Cancer-associated fibroblasts were observed to secrete leptin, which promoted the proliferation and migration of breast cancer [28]. Interestingly, the cooperation of cancer cells with these fibroblasts significantly increased the production of cytokines, inflammatory mediators and growth factors. Furthermore, leptin can interact with transforming growth factor beta (TGF-β), phosphatidylinositol 3-kinase (PI3K), protein kinase B (AKT), JAK (Janus kinase), and signal transducer and activator of transcription (STAT). This indicates its influence on the activity of signaling pathways within the tumor microenvironment, which result in further cancer development [29].

One of the most important signaling pathways with which leptin interacts is the JAK/STAT pathway, the products of which are TNF-α and IL-6 [30]. Enhanced JAK/STAT signaling leads to increased cell survival, proliferation, and drug resistance of many cancers [31], including breast cancer [32], gastric cancer [33], and ovarian cancer [34]. It has therefore been proposed to stop aberrant JAK/STAT activation by using small molecules inhibiting JAK (JAKi). Blocking the leptin-dependent JAK/STAT signal can help counter the excessive proliferation as well as the uncontrolled inflammation that accompanies the neoplastic processes [35].

Leptin’s interaction with its receptor also enables the activation of the mitogen-activated protein kinase (MAPK)/extracellular signal-regulated kinase (ERK) and PI3K/AKT pathways [36]. Li et al. observed that leptin produced by cancer-associated fibroblasts activated both of these pathways in non-small-cell lung cancer [37]. Similarly, in papillary thyroid cancer, leptin increased its ability to migrate [38]. Leptin can also signal through TGFB1 in breast cancer, which promotes metastasis and cancer stem cells behavior. Many of the pathways activated by leptin overlap with those involved in the epithelial-mesenchymal transition (EMT), including PI3K/AKT, MAPK/ERK, TGFB1, and Wnt/β-catenin, which increases the mobility, migration, and invasion potential [39]. Xu et al. observed that leptin induces EMT by activating ERK-mediated signaling and promotes migration and invasion of lung cancer cells [40].

In this study, the expression profile of leptin and its receptors was assessed both at the gene and protein levels, which aimed to present more complex data regarding leptin activity. Moreover, the study was conducted on endometrial tissue samples and whole blood collected from patients. Microarray analysis revealed the overexpression of leptin-related genes in EC samples, which was validated by RT-qPCR. Similarly, with whole blood, an increase in the level of leptin and its receptors was also observed in patients with endometrial cancer. Leptin was reported to be a gene characteristic of G2 and G3 cancer, and its overexpression increased with EC grade. On the other hand, LEPR showed a significant increase in all three grades of endometrial cancer. Interestingly, overexpression of LEPR in colorectal cancer was associated with an increase in metastatic potential and enhanced neoangiogenesis [41]. Zheng et al. proposed leptin knockdown as a new strategy to prevent the progression of lung cancer by blocking the activity of the JAK/STAT3 and Notch pathways [42].

In our study, we also observed the overexpression of the other leptin receptors, i.e., LEPROT and LEPROTL1, which indicated the high activity of leptin detected both in tissue samples and whole blood. In the next stage of the analysis, the leptin concentration in the serum of the patients was determined. A significant increase in this protein level was demonstrated in all grades of endometrial cancer, with the highest in G3. Interestingly, the concentration difference between the control and G1 cancer was more than six-fold. Keeping in mind that obesity is one of the risk factors for endometrial cancer, in this study the correlation between BMI and serum leptin concentration was analyzed. Strong positive correlations were observed, with BMI and G3 EC being the strongest. Our results were consistent with the study by Ma et al., which also reported an increase in the serum leptin level in patients with endometrial cancer [43], and Petridou et al. who found high levels of serum leptin by radioimmunoassay [44]. Furthermore, Zhou et al. observed high levels of leptin and its receptor in endometrial cancer cell lines, with the highest in poorly differentiated cancers [45]. In addition, Wang et al. performed a meta-analysis from which they concluded that a high level of leptin was an independent risk factor for endometrial cancer [46].

Gao et al. conducted studies on 1104 women with endometrial cancer and found that a higher BMI was associated with stage I endometrial cancer. Considering the type of cancer, they observed a negative correlation between BMI and the stage of endometrial cancer. They also suggested that obesity and estrogen levels may be related to the development of type II endometrial cancer, which is also influenced by earlier time of diagnosis, non-Western lifestyle, and ethnicity [47]. It was suggested that Western-style diet leads to increased intestinal bacteria associated with obesity, due to the high amount of fat and refined carbohydrates [48]. Microbial imbalance may also promote the development of cancer [49], including colorectal cancer [50], and affect cancer therapy by regulating drug efficiency [51].

The observed overexpression of leptin and its receptors in our study may be a potential therapeutic target for endometrioid endometrial cancer, as it was previously proposed in lung cancer, however more research is needed. A possible therapeutic strategy is inhibition of signaling pathways over-activated by leptin, which was proposed for ovarian cancer [52] and breast cancer [53]. Carino et al. conducted research on endometrial cancer cell lines (Ishikawa, SK-UT2, An3Ca, primary EEC) and benign endometrium (HES) using JAK/STAT, MAPK/ERK, PI3K/AKT, and mTOR inhibitors to determine how leptin-mediated signaling affected the level of pro-inflammatory and pro-angiogenic cytokines. They found that the effect of leptin was cell-specific and that malignant cells were more sensitive to its stimulatory effect on pro-inflammatory and pro-angiogenic cytokines [54].

Our results are consistent with studies reporting high levels of leptin and its receptors in endometrial cancer. It is worth noting that we performed an analysis at the transcriptome and proteome levels to show the changes in the expression of leptin and its receptors in endometrial cancer. Our results were validated as they are consistent regardless of the method used. In addition, our research was carried out on tissue samples as well as whole blood collected from patients. It would be beneficial to increase the size of the study group, as well as use additional analysis methods such as next-generation sequencing. Considering a number of factors and signaling pathways related to leptin activity, it will be valuable to extend further analysis to its target molecules and to comprehensively analyze their interactions. This would help propose strategies based on leptin knockdown or blocking leptin-related pathways in endometrial cancer.

## 5. Conclusions

The development of new cancer treatment strategies and the identification of novel molecular markers are especially important as cancer incidence is still very high. Obesity, which can contribute to cancer progression, including endometrial cancer, is also a major health problem. Leptin interacts with TGF-β, JAK/STAT, PI3K/AKT, MAPK/ERK, and Wnt/β-catenin signaling, which overlap with epithelial-mesenchymal transition and are important for increasing metastatic potential. Overexpression of leptin and its receptors LEPR, LEPROT, and LEPROTL1 was reported in endometrioid endometrial cancer both at the mRNA and protein levels. These results were obtained in endometrial tissue samples and whole blood collected from patients. Moreover, there were strong positive correlations between patients’ BMI and serum leptin levels. These are important observations that may be useful in designing endometrial cancer treatment strategies.

## Figures and Tables

**Table 1:** Sequence of primers used in RT-qPCR.

mRNA	Sequence
LEP	Forward 5′-GAAGACCACATCCACACACG-3′ Reverse 5′-AGCTCAGCCAGACCCATCTA-3′
LEPROT	Forward 5′-GCTTGGAGAGGCAGATAACG-3′ Reverse ′-AATGTCCTGGGTCCAGAGTG-3′
LEPROTL1	Forward 5′-TGCAATGTGGGAAGAAATGA-3′ Reverse 5′-AAGGAGGAAGCAGAGGAAGG-3′
LEPR	Forward 5′-ACAGTCCCTTTGTGGGTCAG-3′ Reverse 5′-TATCCGAGCTCCAGCGTACT-3′
ACTB	Forward 5′-TCACCCACACTGTGCCCATCTACGA-3′ Reverse 5′-CAGCGGAACCGCTCATTGCCAATGG-3′

RT-qPCR: Real-Time Quantitative Reverse Transcription Reaction; LEP: leptin; LEPR: leptin receptor; LEPROT: leptin receptor overlapping transcript; LEPROTL1: leptin receptor overlapping transcript-like 1; ACTB: β-actine; mRNA: messenger RNA.

**Table 2:** Characteristics of patients enrolled in the study.

Parameter	C	G1	G2	G3
Age (years)	65.36 ± 10.29	67.22 ± 8.04	68.4 ± 10.09	64.88 ± 12.02
Height (m)	1.63 ± 0.14	1.59 ± 0.08	1.62 ± 0.05	1.59 ± 0.04
Weight (kg)	72.99 ± 13.95	74.41 ± 11.79 *#	85.77 ± 21.99 **	85.22 ± 13.11 ***###
BMI	28.77 ± 7.14 (overweight)	29.01 ± 3.14 # (overweight)	36.15 ± 10.44 **## (2nd degree of obesity)	33.18 ± 5.44 ***### (1st degree of obesity)

C, control; G, endometrial cancer grade; and BMI, body mass index. * G1 vs. C (*p* < 0.05); ** G2 vs. C (*p* < 0.05); *** G3 vs. C (*p* < 0.05); # G1 vs. G2 (*p* < 0.05); *##* G2 vs. G3 (*p* < 0.05); *###* G1 vs. G3 (*p* < 0.05).

**Table 3:** Expression profile of LEP, LEPROT, LEPROTL1, and LEPR in the endometrial tissue samples and whole blood of patients determined by microarrays and RT-qPCR (*p* < 0.05; FC > 3 or < −3).

Material	mRNA	ID	Microarray			RT-qPCR		
			G1 vs. C	G2 vs. C	G3 vs. C	G1 vs. C	G2 vs. C	G3 vs. C
Tissue	LEP	207092_at	3.74 *	5.58 *	11.98 *	3.22 *	6.01 *	12.11 *
	LEPROT	202377_at 202378_s_at	4.12 * 4.01 *	4.52 * 4.33 *	4.69 * 5.01 *	3.99 *	4.88 *	4.21 *
	LEPROTL1	202594_at 202595_s_at	8.54 * 8.41 *	7.98 * 8.22 *	9.36 * 10.02 *	9.01 *	8.61 *	9.00 *
	LEPR	209894_at 209959_at 211167_s_at 211354_s_at 211355_x_at	7.58 * 8.01 * 6.58 * 8.14 * 7.69 *	8.99 10.01 * 7.44 * 8.98 * 6.01 *	12.36 * 11.25 * 12.47 * 12.36 * 13.11 *	9.54 *	8.66 *	11.63 *
Whole blood	LEP	207092_at	8.99 *	14.58 *	18.51 *	7.52 *	12.63 *	19.63 *
	LEPROT	202377_at 202378_s_at	5.01 * 4.58 *	4.99 * 5.09 *	4.77 * 5.01 *	4.36 *	4.22 *	4.59 *
	LEPROTL1	202594_at 202595_s_at	7.58 * 7.63 *	8.01 * 8.98 *	10.65 * 11.01 *	8.02 *	8.96 *	10.54 *
	LEPR	209894_at 209959_at 211167_s_at 211354_s_at 211355_x_at 211356_x_at	9.63 * 9.62 * 9.54 * 9.88 * 9.10 * 10.04 *	11.25 * 11.44 * 11.01 * 11.36 * 10.58 * 11.66 *	14.21 * 14.26 * 14.20 * 14.01 * 13.57 * 14.74 *	9.01 *	10.56 *	13.25 *

RT-qPCR—Real-Time Quantitative Reverse Transcription Reaction; LEP—leptin; LEPR—leptin receptor; LEPROT—leptin receptor overlapping transcript; LEPROTL1—leptin receptor overlapping transcript-like 1; ACTB—β-actine; mRNA—messenger RNAID, number of the probe; FC, fold-change; C, control; and G, endometrial cancer grade. * *p* < 0.05 vs. C group.

**Table 4:** Serum leptin concentration in the study and control groups together with the result of the Pearson correlation test (*p* <0.05).

Group	C	G1	G2	G3
ELISA (pg/mL)	58.66 ± 3.63	369.68 ± 2.11 *	499.32 ± 6.18 *	714.01 ± 4.52 *
Pearson correlation (r) BMI	0.889 *	0.841 *	0.914 *	0.924 *

C, control; and G, endometrial cancer grade; ELISA—enzyme-linked immunosorbent assay; r—Pearson correlation; BMI—Body Mass Index; * *p* < 0.05 vs. C group.

## Data Availability

The data used to support the findings of this study is included in the article. The data will not be shared due to third-party rights and commercial confidentiality.

## Supplementary Materials

The following are available online at https://www.mdpi.com/article/10.3390/jcm10132787/s1. Table S1. Exact *p*-values of the expression profile of LEP, LEPROT, LEPROTL1, and LEPR in endometrial tissue samples and whole blood of patients determined by microarrays and RT-qPCR (*p* < 0.05; FC > 3 or < −3).
