# Supplementary material for: Expression Pattern of Leptin and Its Receptors in Endometrioid Endometrial Cancer
Source: J Clin Med. 2021 Jun 24;10(13):2787. doi: 10.3390/jcm10132787 (PMC8268664; doi:10.3390/jcm10132787)
Supplement: Supplementary file 1 [file jcm-10-02787-s001.zip › jcm-1232752-supplementary.pdf]

Journal of
Clinical Medicine

MDPI

Supplementary Material

Table 1. Exact p-value of the expression profile of LEP, LEPROT, LEPROTL1, LEPR in endometrial tissue samples and whole blood of patients determined by microarrays and RT-qPCR ($p < 0.05$; FC> 3 or <-3).

Material	mRNA	ID	Microarray			RT-qPCR		
			G1 vs. C	G2 vs. C	G3 vs. C	G1 vs. C	G2 vs. C	G3 vs. C
Tissue	*LEP*	207092_at	0.0325	0.02294	0.0001	0.0034	0.0018	0.0001
	LEPROT	202377_at	0.0194	0.0155	0.0421	0.03698	0.04125	0.03699
		202378_s_at	0.0369	0.0725	0.0541			
	LEPROTL1	202594_at	0.0001	0.0036	0.0044	0.0025	0.0014	0.0024
		202595_s_at	0.0025	0.0047	0.0047			
	LEPR	209894_at	0.0084	0.0004	0.0001	0.0016	0.0002	0.0001
		209959_at	0.0054	0.0003	0.0001			
		211167_s_at	0.0067	0.0006	0.0001			
		211354_s_at	0.0064	0.0002	0.0001			
		211355_x_at	0.0072	0.0004	0.0001			
Whole blood	*LEP*	207092_at	0.0064	0.0005	0.0001	0.0058	0.0001	0.0001
	LEPROT	202377_at	0.0047	0.0315	0.0364	0.0412	0.0369	0.0354
		202378_s_at	0.0038	0.00954	0.0258			
	LEPROTL1	202594_at	0.0004	0.0009	0.0001	0.0027	0.0008	0.0001
		202595_s_at	0.0039	0.0084	0.0001			
	LEPR	209894_at	0.0001	0.0001	0.0001	0.0001	0.0001	0.0001
		209959_at	0.0001	0.0001	0.0001			
		211167_s_at	0.0001	0.0001	0.0001			
		211354_s_at	0.0001	0.0001	0.0001			
		211355_x_at	0.0001	0.0001	0.0001			
		211356_x_at	0.0001	0.0001	0.0001			

ID, number of the probe; FC, fold-change; C, control; G, endometrial cancer grade. $p<0.05$ vs. C group
